# Plasma Modification of Carbon Coating Produced by RF CVD on Oxidized NiTi Shape Memory Alloy under Glow-Discharge Conditions

**DOI:** 10.3390/ma14174842

**Published:** 2021-08-26

**Authors:** Justyna Witkowska, Michał Tarnowski, Emilia Choińska, Marek Kulpa, Jacek Szade, Gerhard Raugh, Wojciech Święszkowski, Tadeusz Wierzchoń

**Affiliations:** 1 Faculty of Materials Science and Engineering, Warsaw University of Technology, 141 Wołoska St., 02-507 Warsaw, Poland; justyna.aleksandra.witkowska@gmail.com (J.W.); emilia.choinska@pw.edu.pl (E.C.); wojciech.swieszkowski@pw.edu.pl (W.Ś.); tadeusz.wierzchon@pw.edu.pl (T.W.); 2 A. Chełkowski Institute of Physics, University of Silesia, Uniwersytecka 4, 40-007 Katowice, Poland; marek.kulpa@us.edu.pl (M.K.); jacek.szade@us.edu.pl (J.S.); 3 LOT-Quantum Design GmbH, Im Tiefen See 58, 64293 Darmstadt, Germany; rauh@lot-qd.de

**Keywords:** NiTi alloy, glow-discharge treatments, carbon coatings, plasma oxygen modification, wettability, surface free energy

## Abstract

Our previous work has shown that for cardiac applications, combining low-temperature plasma oxidation with an amorphous carbon coating (a-C:N:H type) constitutes a prospective solution. In this study, a short-term modification by low-temperature oxygen plasma is proposed as an example and a method for shaping the topography and surface energy of the outer amorphous carbon coating, produced via the Radio-Frequency Chemical Vapour Deposition (RFCVD) method on NiTi alloy oxidized under glow-discharge conditions. This treatment alters the chemical composition of the outer zone of the surface layer. A slight increase is also noted in the surface roughness at the nanoscale. The contact angles were shown to increase by about 20% for water and 30% for diiodomethane, while the surface free energy decreased by ca. 11%. The obtained results indicate that even short-term contact with low-temperature plasma can shape the surface properties of the carbon coating, an outcome which shows potential in terms of its use in medical applications.

## 1. Introduction

Since it was discovered that a great potential of NiTi shape memory alloy is its use in producing a novel medical implant, its surface modifications have been the interest of research groups across the world, trying to further improve its biocompatibility in terms of its medical applications. However, surface modification is not limited to producing coatings, and can be extended to non-invasive treatments of produced coatings affecting their surface properties. Such hybrid methods that combine a few processes can optimize the final properties of treated materials. When producing a cardiovascular implant, several requirements must be fulfilled because of its contact with blood [1]. Carbon coatings are one of the candidates for cardiovascular implants such as self-extending stents, because of their great hemocompatibility. This means, first of all, that the surface must have atrombogenic properties, i.e., they must prevent the excessive adhesion and aggregation of blood platelets, which lead to the formation of clots and increase the risk of embolism [2]. In addition, the hemocompatible material should be characterized by controlled endothelialization, i.e., the growth of vascular endothelial cells. Endothelialization prevents the phenomenon of restenosis, i.e., the re-narrowing of the vessel, which is a common drawback of angioplasty procedures [3,4]. The material used in the circulatory system must also have the appropriate mechanical properties that allows it to adapt to changing loads (cyclical changes in blood pressure) [5]. The latter requirement is met very well by NiTi alloys, due to their unique properties such as shape memory and superelasticity. Surface properties such as the microstructure, chemical and phase composition, surface topography, and surface morphology require shaping, as they affect surface energy, i.e., the composition of the resulting protein biofilm, which in turn influences the biological properties associated with atrombogenicity and endothelialization [5,6,7,8,9]. NiTi shape memory alloys used to make long-term implants, such as stents, must be able to limit nickel ion release into the surrounding biological environment (metallosis). Such properties are ensured to varying degrees by the surface layers produced on the NiTi alloy, such as: titanium oxides, in particular rutile—TiO_2_, titanium nitride—TiN, silicon oxide—SiO_2_, amorphous hydrogenated carbon doped with nitrogen—a-C:N:H, amorphous hydrogenated silicon carbide—a-SiC:N:H produced via different surface engineering methods, which are often applied on, e.g., stents [10,11,12,13,14,15]. A prospective material solution for the shape memory NiTi alloy in terms of its application for cardiological implants is the deposition of composite layers consisting of an interlayer of titanium oxide—TiO_2_—rutile, produced by oxidation under glow-discharge conditions, and a thin a-C:N:H external coating produced via RFCVD. A layer of titanium oxide TiO_2_ (rutile), the most thermodynamically stable of the titanium oxides, which has a nanocrystalline structure, significantly increases the corrosion resistance of the NiTi alloy [16] and forms a barrier, preventing the release of nickel into the biological environment (metallosis). An additional 20–30 nm-thick outer coating of a-C:N:H improves both the corrosion resistance and the hemocompatibility, i.e., by reducing the adhesion of blood platelets and their agglomerates, and by promoting the adhesion of endothelial cells [4,17,18,19]. The properties of a hydrogenated carbon coating doped with nitrogen can also be shaped by heating it in oxygen plasma under glow-discharge conditions directly after the formation of a composite layer, which alters the wettability of the coating, its surface topography, and thus the free surface energy. These factors have a significant influence on the biological properties of carbon coatings [20]. Shaping the properties of various carbon coatings in the postprocessing stage is a current research problem [21,22,23]. Plasma treatments, including argon [21], oxygen [21,22,24], and sulfur hexafluoride [25] plasma, are used to modify the chemical state of the outer zones of the coatings, thus affecting its properties such as wettability, surface topography [21], and other mechanical [17] or electrical properties [23]. In addition, other materials such as polymers [25,26,27] have been recently modified using the possibility of simple one-step plasma treatments and obtained promising properties such as superhydrophilicity [26] or increased adhesion and uniformity of the coatings [27]). The production of a-C:N:H coatings on TiO_2_ oxide layers made under glow-discharge conditions is beneficial due to the enhanced adhesion of the coatings to an oxidized NiTi substrate [28]. Hence, this article highlights the possibility of shaping the surface properties of the outer carbon coating through treatment by low-temperature glow-discharge plasma, with the use of, e.g., a chemically active oxygen atmosphere. As far as we are aware, we present for the first time the possibility of a new surface treatment combining low-temperature plasma oxidization under glow-discharge conditions with the RFCVD process, and short-term oxygen plasma treatment to shape the properties of produced amorphous carbon coatings, including surface topography, chemical composition, wettability, and surface free energy. Our work brings some new knowledge to light in terms of the possibility of modifying the properties of outer carbon coatings without influencing the bulk material-NiTi alloy, as well as simulating additional processes such as sterilization that are necessary for their use in medical implants and can be performed in plasma conditions. They should be considered a final step of surface treatment, since they are not without significance in terms of their influence on the chemical composition and the surface free energy of the outer zone of the surface layers. Therefore, it is important to clearly understand the impact of even short-term plasma treatment on the surface layers and keep this under precise control.

## 2. Materials and Methods

NiTi shape memory alloy (50.8% at. Ni, Ti balance) was used in this study. Samples of Ø14 mm diameter and 1 mm thickness were mechanically ground using sandpapers of up to 2400-grit and cleansed in acetone using an ultrasonic washer. Low-temperature plasma oxidation processes were carried out using oxygen atmosphere at 290 °C and 1.6 hPa were used in a so-called dynamic vacuum. Carbon coatings were produced on the oxidized surface layers using the RFCVD method (MicroSYS 100, Roth & Rau Microsystems, Hohenstein-Ernstthal, Germany) in an atmosphere of CH_4_+N_2_+H_2_, a pressure of 1 × 10^−2^ hPa and a RF generator power of 600 W for 15 min. After the hybrid process, the composite a-C:N:H+TiO_2_ surface layer was modified by low-temperature oxygen plasma at 100 °C for 5 min.

The microstructure of the produced layers was investigated by means of a High-Resolution Scanning Transmission Electron Microscope (HRSTEM) with bright field and high-angle annular dark field detectors (HAADF) (Hitachi, Tokio, Japan), operating under an acceleration voltage of 200 keV. Samples measuring 15 × 3 × 5 µm were prepared for the HRSTEM using the Focused Ion Beam (FIB) lift-out technique and an Ion Scanning Microscope, and were then thinned to approximately 100 nm using a gallium ion beam with an energy of 40 kV. The linear distribution of oxygen, nitrogen, titanium, and nickel in the surface layers was measured by SIMS (Cameca, Quai des Gresillons, France)—i.e., secondary ion mass spectrometry analysis (Cameca IMS6F).

Nanoindentation measurements were carried out using NanoTest Vantage equipment (Micro Materials, Wrexham, UK) with a Berkovich indenter. All measurements were performed with a controlled load in a range from 0.03 mN to 1 mN. For the hardness and the elastic modulus depth profiles, the data from the experimental ranges were divided into intervals for averaging, and then graphs consisting of error bars were generated. The testing technique combined the conventional Oliver and Pharr unloading curve analysis with the rapid acquisition of test data [29].

In order to determine the effect of plasma oxygen treatment on the surface topography of the composite surface layer, a Wyko NT 9300 optical profilometer was used and Atomic Force Microscopy (AFM) observations were conducted using an atomic force microscope (Veeco, Plainview, NY, USA) with a Multimode VIII controller (tapping mode, tip model ACSTA, AppNano) (Applied Nanostructures, Inc., Mountain View, CA, USA).

The chemical composition of the composite layers after the hybrid process, before and after the short-term plasma treatments, was assessed via the X-ray photoelectron spectroscopy (XPS) method, for which a PHI 5700/660 Multifunctional Photoelectron Spectrometer (Physical Electronics, Chanhassen, MN, USA) was used. The assessment also employed a monochromatic beam from an X-ray tube with an Al anode. The energy resolution was 0.2 eV.

The wettability test was performed using an OCA 20 Contact Angle System goniometer (DataPhysics Instruments, Filderstadt, Germany) at room temperature for two liquids: distilled water and diiodomethane. A droplet of each liquid (0.4 μL) was discharged on the surface of each sample and its image was captured immediately after stabilization. Measurements for each liquid were performed at least 10 times and the average values and standard deviations were calculated. For analysis of the profiles, SCA 20 software (DataPhysics Instruments, Filderstadt, Germany) was used. Depending on the obtained values of the contact angles for distilled water and diiodomethane, the surface free energy was calculated for each sample using the Owens–Wendt standard method [30].

The properties (including the biological properties) of the composite a-C:N:H+TiO_2_ surface layer, produced in a hybrid process combining oxidation under glow-discharge conditions and RFCVD, were presented in our earlier studies [31], which revealed that its outer zone is formed of amorphous carbon.

## 3. Results and Discussion

**Figure 1:**
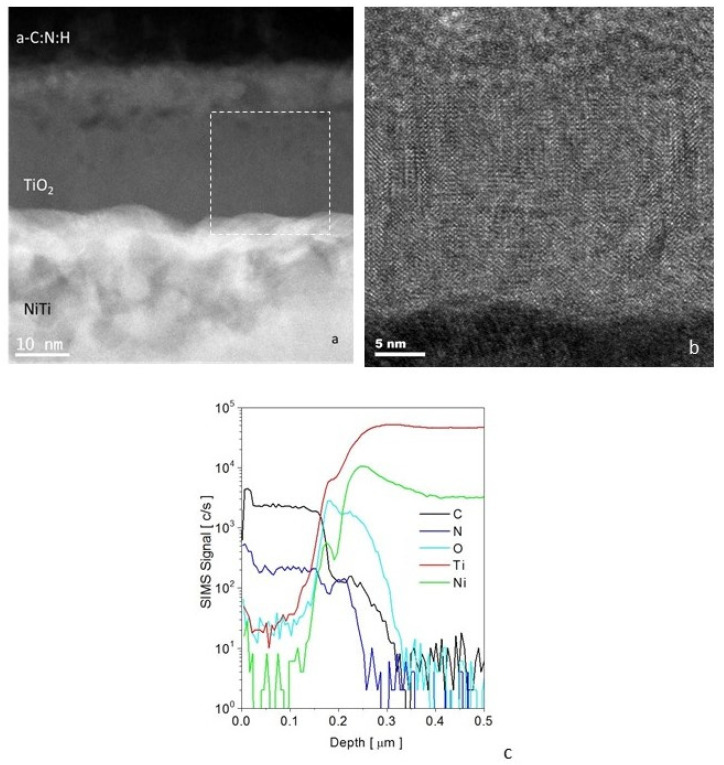
Microstructure of an a-C:N:H + TiO_2_-type composite layer produced on NiTi alloy in a hybrid process: (**a**) STEM image of composite layer, (**b**) HRTEM TiO_2_ sublayer, and (**c**) distribution (SIMS) of carbon, oxygen, nitrogen, nickel, and titanium in a-C:N:H+TiO_2_ surface layer.

Transmission Electron Microscope (TEM) images show the microstructure and chemical composition of the composite layer produced using a hybrid method, combining glow-discharge oxidation and RFCVD (Figure 1a).

The outer zone of the layer consisted of a 40 nm coating of hydrogenated amorphous carbon doped with nitrogen (a-C: N: H) and was produced on a 30 nm titanium oxide-TiO_2_ (rutile) layer with a nanocrystalline structure (Figure 1b). As shown in Figure 1, the structure of titanium oxide in the zone located directly under the carbon coating was mixed—amorphous and nanocrystalline with nanopores and with a developed surface, resulting in the enhanced adhesion of the a-C:N:H coating.

**Figure 2:**
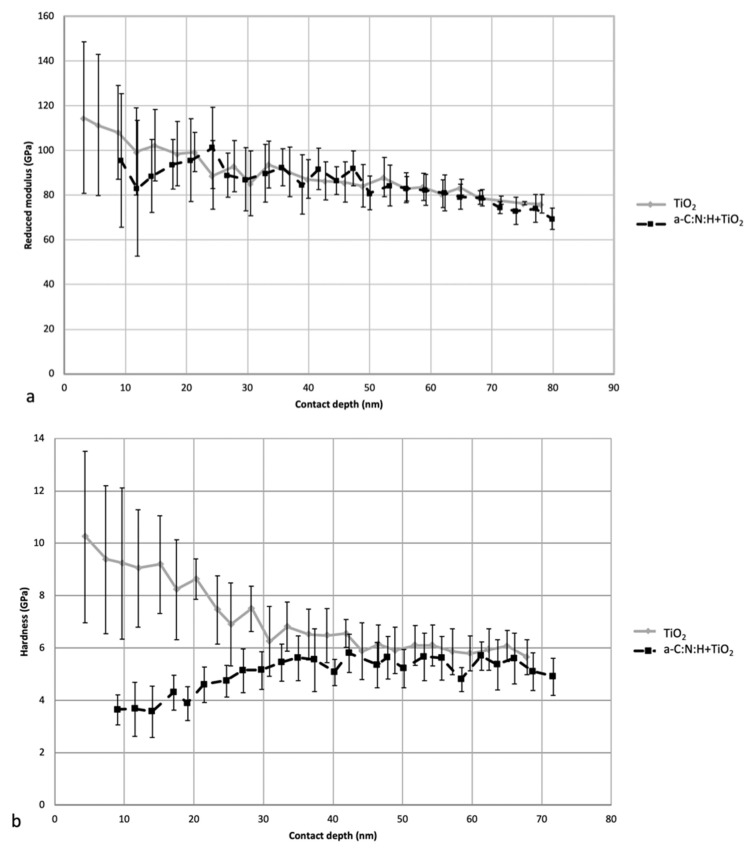
Changes in reduced modulus of elasticity (**a**) and hardness (**b**) with contact depth of the indenter for samples with an oxidized layer and an oxidized layer with a carbon coating.

The TiO_2_ layer featured increased hardness—up to 10 GPa—and a higher reduced modulus of up to 118 GPa, at depths indicating the presence of a layer. These values were higher compared to those stabilized at a greater depth, i.e., at the core values (NiTi alloy), which were 5 GPa and 80 GPa, respectively (Figure 2). The instability of the results for small depths resulted from the surface roughness and from the porous amorphous and nanocrystalline structure of the TiO_2_ outer zone. The hardness and reduced modulus of the elasticity values decreased with the indentation depth and stabilized at 40–50 nm. The formation of a coating of hydrogenated amorphous carbon doped with nitrogen (a-C:N:H) lowered the hardness and reduced the modulus of the elasticity of the outer layer, with stabilization at a similar level.

**Figure 3:**
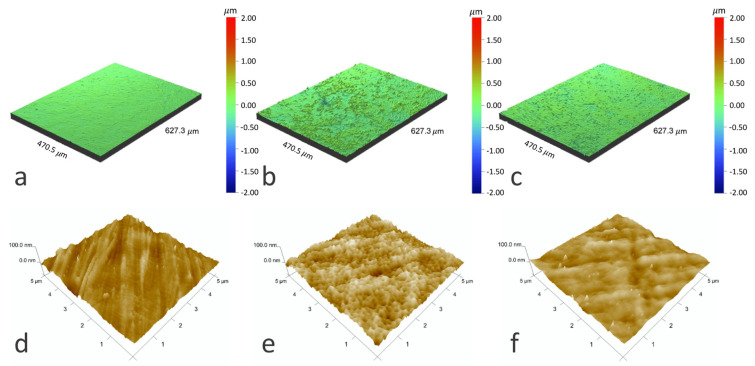
Optical profilometer images (**a**–**c**) and AFM images (**d**–**f**) of NiTi alloy surface in its initial state (**a**,**d**), after glow-discharge oxidization and RFCVD process (**b**,**e**) and after glow-discharge oxidization and RFCVD process followed by short-term modification by oxygen plasma (**c**,**f**).

Surface topography (Figure 3, Table 1) measurements showed an increased surface roughness after the hybrid process at the microscale, while the surface roughness at the nanoscale was similar to that of the NiTi alloy in its initial state. Differences in the effect of short-term treatment by oxygen plasma were observed. At the microscale, the oxygen plasma made the surface slightly less rough compared to the samples with an a-C:N:H+TiO_2_ layer before treatment. At the nanoscale, plasma treatment increased the surface roughness, but the differences were much smaller. Most researchers observed that plasma leads to an increase in roughness parameters [32], the related surface area, and the amount of surface defects of some carbon structures such as fibers [33,34] or nanotubes [35]. However, they also indicate that an extended treatment duration can result in the opposite effect—smoothening of the surface [34].

**Figure 4:**
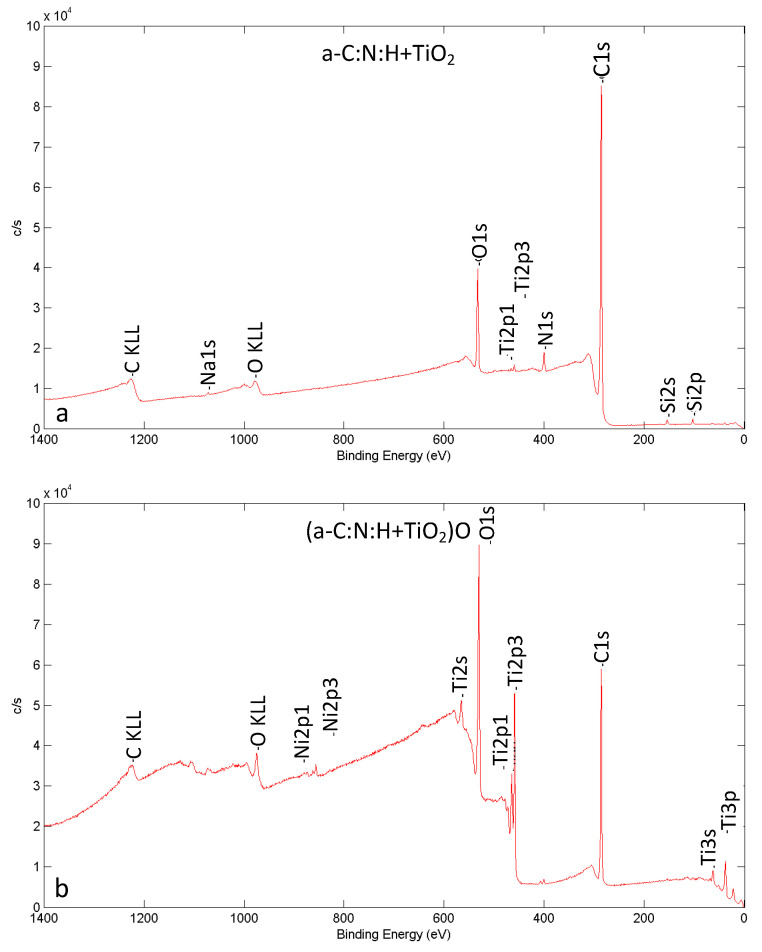
XPS spectra for NiTi alloy surface after glow-discharge oxidization and RFCVD before (**a**) and after short-term modification by oxygen plasma (**b**).

The chemical composition of the composite surface layer’s outer zone changed after short-term plasma treatment (Figure 4, Table 2). The oxygen content in the layer increased, while the carbon content dropped. The increased values of Ti and Ni content can indicate that the there was a reduction in the layer thickness during plasma modification. The presence of small amounts of Si can be considered a side effect of the sample preparation. The spectra for all the analyzed elements lead us to conclude that the chemical state and functional groups in the outer zone of the composite layer were altered after the short-term plasma modification.

The changes in chemical composition after plasma treatment correspond to the literature findings, which indicate an increase in oxygen content and a decrease in carbon content on the surface after plasma treatment [33,36,37,38,39], a correlation also referred to as the O/C ratio. This is because the oxygen plasma treatment causes the carbon–carbon bonds to break [36,38] resulting in the incorporation of functional groups, which include C–O, O–C–O and C=O [36,39], into the surface.

The contact angles increased after short-term plasma treatment for both the tested liquids (Table 3). According to calculations based on the Owens–Wendt method, surface free energy was shown to decrease after short-term treatment, and was similar to that calculated for the NiTi alloy in its initial state. We observed that short-term oxygen plasma treatment approximates the values of contact angles to those obtained for the oxidized layer itself, whereas the values obtained for the carbon coating before plasma modification are similar to those in the initial state. The higher contact angles for the samples after plasma treatment are not typical for those reported in the literature, which point to a decrease in contact angles of different carbon surfaces after plasma treatment [35,36,40].

## 4. Conclusions

The aim of the study was to propose a novel surface modification method for the NiTi shape memory alloy that will be beneficial in the context of medical applications, especially as cardiological implants. The proposed modification consists of the improvement of a hybrid process which combines oxidization at low-temperature glow-discharge plasma with the RFCVD process enabling the production of amorphous carbon coatings, by the addition of a step of short-term treatment of the produced a-C:N:H + TiO_2_ layers in the oxygen plasma. The greatest advantages of the described processes include, first of all, the use of a low temperature and the short duration of the processes at each stage, as well as the possibility of shaping surface properties in the nano scale, which is of key importance for the interaction of the surface with its biological environment.

It is worth noting that the use of temperatures below 300 °C during the glow-discharge processes made it possible to produce surface layers that guarantee the preservation of the specific properties of the NiTi alloy, i.e., shape memory and superelasticity, which was previously demonstrated on the example of nitrided (TiN) or oxynitrided (TiO_2_+TiN) layers with thicknesses of several dozen nanometers, produced at various temperatures [41]. Moreover, the nitrided and oxynitrided layers of the nanocrystalline structure with thicknesses of several dozen nanometers did not show any cracks or damage after the shape recovery tests [41], mainly due to their nanocrystalline structure and small thickness. This is very important in the context of applications for implants using the shape memory phenomenon (e.g., self-expanding stents). The presented research results and their analysis in the context of NiTi alloy applications in cardiology support the use of the NiTi alloy treatment processes in low-temperature glow-discharge plasma conducted at a temperature below 300 °C, and in a timeframe that allows for the production of oxide layers with the thickness of several dozen nanometers, which are a suitable basis for the production of the thin amorphous carbon a-C:N:H coatings. Additional short-time modification of that carbon coating is an innovative supplement to the proposed hybrid process, allowing for slight changes in the surface topography and chemical composition of the surface layers, which changes their properties, including biological properties.

Plasma treatment of various types of carbon materials is an effective way of shaping their surface properties without affecting the bulk of the material. According to the literature, this method has so far been used to modify the surface of carbon fibers, carbon nanotubes, activated carbon, carbon black, glassy carbon, graphite, diamonds, and amorphous carbon. Different gas atmospheres, such as oxygen, hydrogen, air, nitrogen, argon, trifluoromethane, etc., and different process parameters including temperature, time of treatment, etc., are used to yield the desired effect [21,22,23,24,25,26]. The short-term low-temperature oxygen plasma modification of the presented a-C:N:H amorphous carbon coating deposited on the plasma-oxidized NiTi shape memory alloy yielded slight changes in the surface topography at the micro- and nano-scale, changes in the chemical composition of the surface, and resulted in decreased wettability, making it a viable technique for altering the biological properties of such surface layers. It should be emphasized that a composite layer with a homogeneous structure of this type can be deposited on complex-shaped parts made not only of NiTi alloy, but also of titanium and its other alloys, which is of great significance in implantology. Therefore, it is a potential method for medical applications in particular, as it can be used to shape the surface parameters of the produced surface layers without influencing their microstructure.

## Figures and Tables

**Table 1:** Results of surface roughness measurements with the use of an optical profilometer and AFM.

Method	Sample	R_a_ [nm]	R_q_ [nm]	R_t_ [nm]
Optical profilometer	NiTi	22 ± 3	30 ± 2	1321 ± 226
	a-C:N:H+TiO_2_	63 ± 16	87 ± 24	2243 ± 504
	(a-C:N:H+TiO_2_)O	52 ± 18	70 ± 2	1157 ± 7
AFM	NiTi	10 ± 2	15 ± 3	188 ± 34
	a-C:N:H+TiO_2_	11 ± 2	14 ± 3	196 ± 93
	(a-C:N:H+TiO_2_)O	14 ± 7	21 ± 12	214 ± 137

R_a_—arithmetic average of absolute values of profile heights over evaluated length, R_q_—root mean square average of the profile heights over evaluated length. R_t_—distance between the highest and the lowest point of the measured section.

**Table 2:** Results of XPS investigations of NiTi alloy surface after glow-discharge oxidization and RFCVD before and after short-term modification with oxygen plasma.

Elemental Composition of the Surface (XPS)									
% at.	C	O	N	Ti	Ni	Si	rest	O/C	N/C
a-C:N:H+TiO_2_	83.3	11.3	3	0.2	0.1	1.3	0.8	0.14	0.04
(a-C:N:H+TiO_2_)O	51.6	34	1	11	0.7	0.5	1.2	0.66	0.02
	a-C:N:H+TiO_2_				(a-C:N:H+TiO_2_)O				
Line	Bonding energy (eV)	FWHM (eV)	Chemical state/bonds	Atomic %	Bonding energy (eV)		FWHM (eV)	Chemical state/bonds	Atomic %
C1s	285.3	1.5	C-C	71.7	285.4		1.2	C-C	46.4
C1s	286.9	1.6	C-N	8.3	286.5		1.3	C-N	3.6
C1s	288.8	2	C-O	3.3	288.8		1.7	C-O	1.6
N1s	399	1.6	pyridinic-N. N-C	0.9	399		1.3	pyridinic-N. N-C	0.1
N1s	400.3	1.9	pyrrolic-N. N-C	1.9	400.3		1.9	pyrrolic-N. N-C	0.3
N1s	402.7	2.1	pyridine-N-oxide. N-C	0.2	402.3		1.9	pyridine-N-oxide.	0.1
								N-C	
N1s	-	-	-	-	406.9		1.3	nitrates. -NO_3_	0.4
O1s	-	-	-	-	530		1.2	TiO_2_	23.1
O1s	531.8	1.6	O-C	1.9	531.1		1.6	O-C	5.1
O1s	532.9	1.8	SiO_2_. O-Si	2.6	533		1.8	SiO_2_. O-Si	1.1
O1s	532.9	1.8	OH. O-H	4.5	533		1.8	OH. O-H	3.5
O1s	534.2	1.6	O-H-O	2.3	533		1.8	nitrates	1.2
Ti 2p	-	-	-	-	458.8		1.2	TiO_2_	11.2
Ti 2p	-	-	-	-	464.4		2.1	TiO_2_	1.8
Si2p	103	1.5	SiO_2_. Si-O	1.3	103		1.5	SiO_2_. Si-O	0.5

**Table 3:** Wettability and surface free energy of NiTi alloy surface in initial state, after glow-discharge oxidization, after glow-discharge oxidization and RFCVD, and after glow-discharge oxidization and RFCVD followed by short-term modification by oxygen plasma.

		NiTi	TiO_2_	a-C:N:H+TiO_2_	(a-C:N:H+TiO_2_)O
Contact angles (°)					
water	mean	83.09	106.61	84.22	101.79
	sd	4.26	0.90	1.78	1.30
diiodomethane	mean	83.09	106.61	84.22	101.79
	sd	4.26	0.90	1.78	1.30
Surface Free Energy					
γ	[mN/m]	36.37	25.78	38.4	34.17
γ_d_	[mN/m]	33.69	25.78	36.58	34.17
γ_p_	[mN/m]	2.67	0.00	1.82	0

## Data Availability

The data presented in this study are available on request from the corresponding author.
